# Free-Standing Nanopatterned Films of Silk Sericin and Gellan Gum and the Spectroscopy Studies from Eu^3+^-Doped Films

**DOI:** 10.1021/acsomega.5c03998

**Published:** 2025-10-25

**Authors:** Francisco R. Torres, Roberta S. Pugina, Leticia de Oliveira, Molíria V. dos Santos, Hernane S. Barud, Sidney J. L. Ribeiro, José Maurício A. Caiut

**Affiliations:** † Grupo de Nanomateriais e Sistemas Luminescentes, Departamento de Química, Faculdade de Filosofia, Ciências e Letras de Ribeirão Preto, Universidade de São Paulo (FFCLRP-USP), Ribeirão Preto, São Paulo 14040-900, Brazil; ‡ Instituto de Química, 28108 Universidade Estadual Paulista (UNESP), Araraquara, São Paulo 14800-900, Brazil; § Faculdade de Engenharia Química, 28132 Universidade Estadual de Campinas (UNICAMP), Campinas, São Paulo 13083-852, Brazil; ∥ BioSmart Nanotechnology Ltda, Araraquara, São Paulo 14808-162, Brazil; ⊥ Laboratório de Biopolímeros e Biomateriais, Universidade de Araraquara (Uniara), Araraquara, São Paulo 14801-340, Brazil

## Abstract

In this study, we investigated the development of flexible and nanopatterned hybrid films composed of silk sericin (SS) and gellan gum (GG) for photonic applications. GG is widely recognized for its ability to form free-standing films with excellent transparency and flexibility, whereas pristine SS films often exhibit limited mechanical and optical performance. By combining SS and GG, we engineered composite films with improved functionality while maintaining high transparency, unlocking new possibilities for diverse applications in biophotonics. Here, soft lithography was employed to fabricate transparent nanostructured SSGG composite films with different geometries. Additionally, we successfully fabricated high-quality, red-emissive optical SSGG composite films by incorporating Eu^3+^ ions at low concentrations. A comprehensive characterization of the composite film’s structure was achieved through an integrative approach, combining scanning electron microscopy, atomic force microscopy, vibrational spectroscopy, and thermogravimetric analyses. The interaction between the biopolymer-based films and lanthanide ions was explored, revealing significant modifications in their spectroscopic profile, particularly in the excitation process. We present a possible energy transfer mechanism from biopolymers to the Eu^3+^ ions and discuss the ^5^ *D*_0_ excited-state lifetime, suggesting coordination within either hydrophilic or hydrophobic environments. Our findings demonstrate that the spectroscopic behavior of the films varies with the preparation method, highlighting the potential for tuning optical properties through material design. These results provide new insights for applications in sensors, smart materials, and optical devices, expanding the scope of biobased photonics.

## Graphical abstract

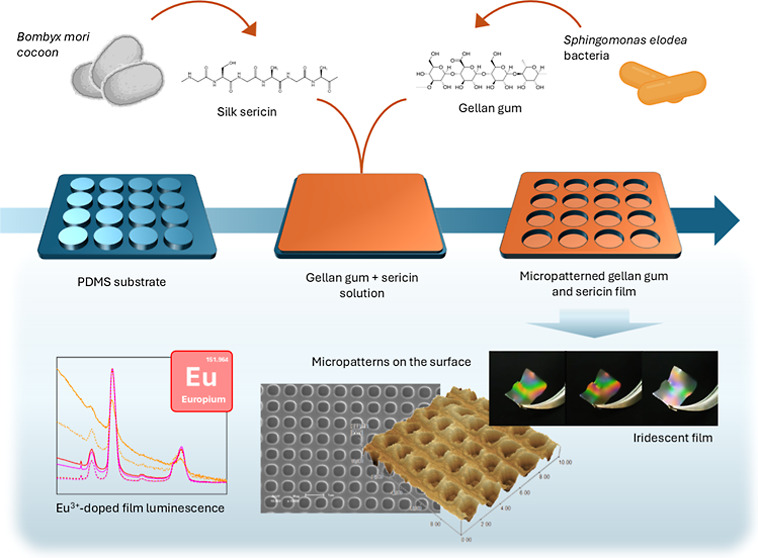

## 1 Introduction

Over the past few decades, the fabrication of spatial patterns using natural polymers has gained significant attention due to their biocompatibility, mechanical properties, biodegradability, renewability, and diverse functionalities. These micro- and nanopatterned structures have found widespread applications in cell biology, pharmaceutical sciences, soft photonics, tissue engineering, and bioelectronics. Several natural polymers, including cellulose, chitin, silk, alginate, collagen, and keratin, have been extensively explored for such applications.^1–6^

Silk is a natural protein produced in the form of fibers by some invertebrates that offers a wide range of applications in biotechnology and photonics. Considering *Bombyx mori*, silk is produced in specific glands in the manufacture of a cocoon, composed of fibroin (SF) (∼70%), a protein from the fibrous class that provides resistance to the cocoon, and sericin (SS) (∼30%), an adhesive protein that joins the SF fibers and protects the cocoon.^7^ SF, recognized as one of the strongest and most resilient natural fibers, has been widely studied for applications across diverse fields due to its biocompatibility and structural properties.^7–15^ However, during the SF extraction process, SS is typically discarded,^16^ despite exhibiting promising properties for photonic applications that remain largely unexplored.^17^ Recent studies have highlighted SS in antioxidant and bactericidal compounds,^18–20^ cosmetic formulations,^21^ and food industry applications.^16,17^ More importantly, the unique protein structure of SS provides an opportunity to develop photonic devices. Systems capable of responding to environmental changes, such as humidity, temperature, gas concentration, and pH, can be designed using structural color phenomena.^22–25^

Gellan gum (GG), on the other hand, is a water-soluble polysaccharide produced by the bacterium *Sphingomonas elodea*. It consists of a linear structure composed of glucose, rhamnose, and glucuronic acid units making it highly versatile for various applications.^26^ GG exhibits high mechanical strength and is resistant to syneresis (water expulsion from gels) and tunable viscoelastic behavior, which can be modified by blending with other polymers or adjusting ion concentrations.^27^ As a nontoxic, biodegradable alternative to synthetic polymers, GG reduces environmental impact and is being explored for use in biosensors, coatings, and packaging.^28^ Additionally, GG has demonstrated the ability to form highly biocompatible hydrogels and self-supporting transparent films. Despite these advantages, its potential for optical applications remains largely underexplored, presenting exciting opportunities for material science and technological advancements.

In this work, we demonstrated the preparation of free-standing films based on SS and GG, which exhibit a synergistic interaction, where GG plays a key role in enhancing the mechanical strength and optical performance of SS, overcoming its inherent limitations. Furthermore, these films exhibit the ability to replicate the surface of the substrate on which they are prepared, making them promising for applications in biomimetic materials,^29^ coatings,^28^ and patterned surfaces.^30^ In addition, our approach demonstrates the feasibility of incorporating lanthanide ions into these composite films and tunes their spectroscopic profiles while preserving their intrinsic properties. Specifically, the lanthanide ions were interesting, because the well-known spectroscopy properties resulted from intraconfigurational 4f^*n*^ transitions could be a strategy to produce new materials. As resulted from a lanthanide electronic structure, different energy conversion mechanisms may occur from materials based on Ln^3+^ ions, such as downshifting, downconversion, and upconversion. So, these spectroscopy properties may be useful for new applications. Herein, doping the hybrid material with Eu^3+^ ions is a strategy to study the structural changes at the molecular level, since this ion is a recognized structural probe,^31,32^ and the coordination changes can be monitored by luminescence analysis. These materials exhibit high tunability for diverse applications, ranging from luminescent materials to optical sensors, reinforcing their potential for future photonic devices.

## 2 Experimental Section

### 2.1 Materials

SS was obtained by extracting cocoons of the silkworm (*B. mori*) supplied by the company Fiação de Seda BRATAC S.A. (Londrina/PR, Brazil). Low-Acyl Gellan Gum (LAGG) sample Kelcogel CG-LA (Lot. 0H6717A) was provided by CP Kelco (Limeira/SP, Brazil) and used without further purification. For all processes, high-purity deionized water (18.2 MΩ/cm) obtained from a Millipore Milli-Q water purification system was used. Europium chloride (EuCl_3_·6H_2_O) and gadolinium chloride aqueous solution (GdCl_3_·6H_2_O), used for materials doped with Eu^3+^ and Gd^3+^ ions, were obtained by hydrochloric acid digestion (HCl, Sigma-Aldrich) of europium­(III) oxide (Eu_2_O_3_, Sigma-Aldrich) and gadolinium­(III) oxide (Gd_2_O_3_, Sigma-Aldrich), respectively.

### 2.2 Production Methods

SS was extracted by HTHP treatment.^33^ Nine grams of silkworm cocoons were cut into small strips and immersed in 300 mL of deionized water in a flask, which was treated in a laboratory autoclave for 1 h at a temperature of 120 °C and a pressure of 1.0 kgf/cm^2^. After this degumming process, the SS, now in solution, was separated from the fibroin fibers by filtration and concentrated at 12 mg/mL.

#### 2.2.1 Preparation of GG/SS Composite Films

SS solution was used for the preparation of composite films with GG. The precursor solution for each composite film was prepared by keeping the total (polysaccharide and protein) mass at 200 mg, just varying the proportions of GG and SS. For each formulation, a 2% (w/v) LAGG solution was initially prepared, dispersing the corresponding amount in water at 70 °C under stirring (400 rpm), maintaining for 15 min after dissolution.^34^ Then, the temperature was reduced to 40 °C before addition of the required volume of the SS solution. The mixture was kept under stirring for more than 15 min. The final solutions were placed in polystyrene Petri dishes (diameter = 5.5 cm) and dried at 30 °C for 24 h to obtain the films. The samples were named GGSS_XY, where *X* and *Y* are the percentage of GG and SS, respectively. For example, a film of 75% (w/w) GG and 25% (w/w) SS was named GGSS_7525. The LAGG-only film was prepared without the addition of SS (GG100), and the SS film was prepared only by drying the initial solution (SS100).

#### 2.2.2 Preparation of Structured GG/SS Composite Films by Soft Lithography

The composite solutions prepared in Section 2.2.1 were used to obtain structured films by lithography; so, these solutions were also dried in an oven at 30 °C for 24 h on two different polydimethylsiloxane (PDMS) substrates, called template *C* and *G*. Each one shows different structured patterned surfaces to perform soft lithography to form micropatterns in the final film, and the obtained samples were named with the ending _*C* and *_G*, respectively.

#### 2.2.3 Preparation of Ln^3+^-Doped GG/SS Composite Films

The volume of aqueous solution of LnCl_3_ corresponding to the intended doping in percentage by mass, 0.4%, 2%, and 4% (w/w, ion weight/SS weight) for Eu^3+^ and 2% (w/w) for Gd^3+^, was added under stirring at the concentrated SS solution. Samples were named SSXLn, whereas X is the Ln^3+^ concentration in % (w/w), and Ln corresponds to the specific ion. The GGSS_5050 composite was also doped with 1% Eu^3+^ (w/w), based on total (polysaccharide and protein) mass, following two different routes. In the first route, the required volume of the aqueous solution of Eu^3+^ was added to the aqueous solution of GG and this medium was kept under stirring; then, the required volume of aqueous solution of SS was added. The resulting sample was named Eu_GGSS. In the second route, the Eu^3+^ aqueous solution was added initially into the SS solution, and finally, the required volume of the GG aqueous solution was added; this sample was named GGSS_Eu. These routes were proposed to analyze the preferential coordination from the Eu^3+^ ion on these biological matrices.

### 2.3 Characterization Methods

The optical transmission spectra of the films were obtained with a Thermo Scientific Evolution 60 S UV–vis Spectrophotometer, in a range of 200 to 1100 nm. The vibrational spectroscopy was carried out in attenuated total reflectance (ATR) mode at room temperature on a Bruker Vertex 70 FTIR, in the range of 4000 to 400 cm^–1^, with the accumulation of 40 scans. Raman spectra were obtained using the XploRA PLUS micro-Raman spectrometer (HORIBA France SAS) with a 532 nm green laser and a ×50 VIS-LWD-DF objective lens. The film samples placed on a glass slide and the spectra were collected in the range of 700 to 1900 cm^–1^, with 10 accumulations and 10 s time for each accumulation, 1800 gr/mm grating, and fluorescence removal correction. Thermogravimetric analyses were carried out in a TA Instruments Q600-SDT, under nitrogen flow (100 mL/min) and heating ramp from 10 °C/min to 700 °C. The evaluation of mechanical properties was carried out on an Instron Universal Testing Machine model 5569, with a load of 5 N and a rate of 5.0 mm/min, and Young’s modulus values were a mean and deviation of three measurements. Scanning Electron Microscopy (SEM) was performed on film samples fixed with carbon tape onto aluminum sample holders and sputter coated with gold using a Bal-Tec SCD 050 Sputter Coater. A Shimadzu SS-550 microscope was used in a high-vacuum mode and secondary electron (SE) contrast method, at an accelerating voltage of 10 kV and magnifications from 1000 to 10000×. The film surface was investigated by an Atomic Force Microscope (AFM) on a Shimadzu SPM-9600 microscope, in air at room temperature, in intermittent contact mode (Phase mode), using silicon tips Nanosensors PPP-NCHR. *R*_a_ and *R*_z_ values were given from analysis of eight different regions of the sample. Photoluminescence data were obtained at room temperature and at 77 K using a Horiba Scientific FluoroLog 3 spectrofluorimeter (FL3-122) equipped with a dual excitation and emission monochromator and a Hamamatsu R928 photomultiplier. A 450 W continuous xenon lamp was used to collect excitation and emission spectra and a pulsed lamp for lifetime measurements. All Eu^3+^ ion emission parameters, as total spontaneous emission coefficient (*A*_Total_), the Judd–Ofelt intensity parameters (Ω_λ_), the number of water molecules (*q*) in the Eu^3+^ ion coordination sphere, the radiative lifetime (τ_rad_), and finally, quantum efficiency (η), were determined following the expressions adapted from Carlos et al.,^35^ eq 1:

1 A0→J=A0→1.I(D0→05FJ07)I(D0→05F107).υ̅0→1υ̅0→J

where (^5^ *D*_0_ → ^7^ *F*_*J*_) is the integrated intensity. The ^5^ *D*_0_ → ^7^ *F*_1_ transition is purely magnetic dipolar and its radiative rate does not depend on the local field imposed by the environment; so, this transition could be used as an intensity standard to obtain the spontaneous emission coefficient from each Eu^3+^ transition.^36^ The radiative transition probability to the transition ^5^ *D*_0_ → ^7^ *F*_1_ (*A*_0–1_) could be determined by eq 2:

2 $$A_{0\to 1}=\frac{4e^{2}{\mathrm{\upsilon ̅}}^{3}}{3\hbar c^{3}}n^{3}D_{MD}$$

where *ℏ* is the Planck Constant under 2π, ${\mathrm{\upsilon ̅}}_{0\to J}$ is the barycenter of the transition, *e* is the elementary charge, *n* being the medium’s refractive index (here, *n* = 1.5), and the oscillator strength has been calculated from the theory as *D*_MD_ = 9.6 × 10^–42^ esu^2^ cm^2^,^37^ resulting in *A*_0–1_ = 14.65 *n* ^3^ in s^–1^.

The quantum efficiency (η) of the samples was obtained through the ratio of τ_exp_ to τ_rad_ (τ_rad_ = 1/*A*_Total_). Finally, the Judd–Ofelt intensity parameters Ω_λ_ (λ = 2, 4, or 6) were calculated applying eq 3:

3 Ωλ=3ℏ.A0→J64π4.e2.υ̅0→J3.χ.<FJ07∥U(λ)∥D005>2

where *ℏ* is the Planck Constant under 2π, *e* is the elementary charge, $A_{0\to J}$ is the Einstein Coefficient related to ^5^_0_ *D*_0_ → ^7^_0_ *F*_*J*_ transition, χ is the Lorentz Factor, ${\mathrm{\upsilon ̅}}_{0\to J}$ is the barycenter of the transition, and <FJ07∥U(λ)∥D005>2 is a matrix element obtained from the literature.^36^

## 3 Results and Discussion

### 3.1 Structural Characterization

In this work, self-supporting films were achieved from 25% to 75% of SS. Characterizations of composites were carried out to elucidate the structural balances and the cooperative properties between these matrices. The UV–vis transmittance spectra (Figure 1a) showed higher visible transparency to films with increasing GG content, an inherent characteristic from this polysaccharide matrix. The average visible transmittance values range from 80% (GGSS_9515) to 75% (GGSS_7030), from 380 to 750 nm. On the other hand, films obtained with 50% or more sericin presented a lower transmittance, between 60% (GGSS_5050) and 55% (GGSS_2575). In addition, the transmittance behavior decreases at a greater rate from 400 nm downward, and these samples presented a yellowish color, characteristic of sericin. In the ultraviolet range, the low transmittance was a result of a cooperative effect from both structures (Polysaccharide and Protein), e.g., the SS shows absorption bands at 280 and 214 nm associated with the aromatic groups of amino acids and peptide bonds, respectively.^38,39^ For samples with high GG concentration, the band shifts slightly up to a lower wavelength, due to an absorption band at 260 nm attributed to the glucuronic acid group in the polysaccharide structure.^40^

**Figure 1:**
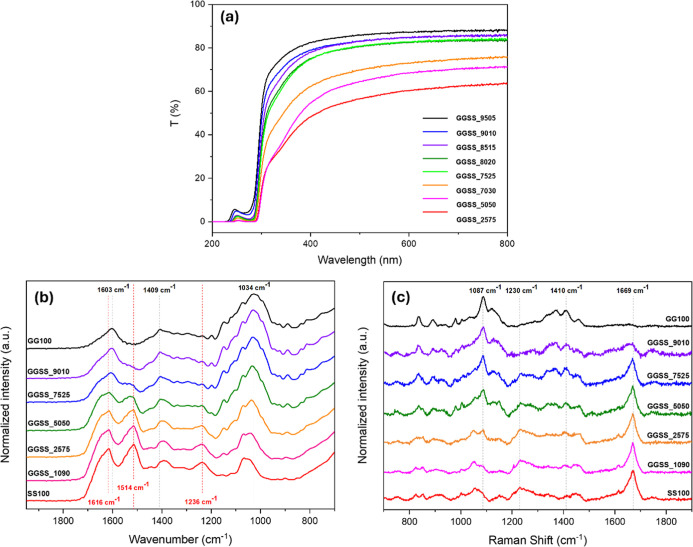
(a) Transmittance spectra in the UV–visible region, (b) vibrational spectra in the infrared region, and (c) Raman scattering spectra of the composite and pristine films of GG and SS.

Vibrational spectra in the infrared region (Figure 1b) are shown for composites and pristine films of each matrix. In the samples containing SS, characteristic bands of the protein were observed,^41^ such as the absorption of amide I represented by the C=O stretching vibration of the amide group at 1616 cm^–1^ and 1637 cm^–1^, the absorption of amide II with contributions from the N–H bending vibration at 1514 cm^–1^ and amide III which arises from C–N stretching vibrations at 1236 cm^–1^. These bands provide information about the conformation of the protein, and such vibrational modes indicate a secondary structure of the SS predominantly of the intermolecular hydrogen-bonded β-sheet.^33,42^ Bands characteristic of the vibrational groups present in the GG structure were identified,^43^ such as at 2925 cm^–1^ referring to the C–H stretching vibration of the CH_2_ group, at 1603 cm^–1^ and 1409 cm^–1^ identified as asymmetric and symmetric stretching of the carboxylate group, respectively, and an intense band at 1034 cm^–l^ attributed to C–O stretching vibrations. The broad band referring to the O–H stretching vibration of the hydroxyl groups, present in the structures of GG and SS, was located at 3600–3200 cm^–1^, with a maximum absorption at 3380 cm^–1^, overlapping and making observation difficult of the N–H stretching vibration band of the amide group at 3500–3000 cm^–1^ expected for materials with SS. It was noticed in the proportion range of the composite films, the characteristic bands of each predominant matrix standing out, of each complementary matrix attenuating. The FTIR spectra of the Eu^3+^-doped composite samples were like those without the ions, regardless of the preparation method.

The Raman scattering spectra of the pristine and composite films samples are shown in Figure 1c. The amide I (1600–1700 cm^–1^) and amide III (1200–1300 cm^–1^) bands are conformation sensitive and may indicate the secondary structure of globular proteins. The data from sample SS100 show a prominent Raman-active band at 1669 cm^–1^ corresponding to the amide I conformation, consisting mainly of a carbonyl (C=O) stretching vibration, and another at 1230 cm^–1^ corresponding to the amide III conformation, consisting of a C–N – H bending vibration and a C–N stretching vibration.^44,45^ These vibrational modes are characteristic and reveal a predominantly β-sheet structure of SS,^46^ which corroborates the assessment from infrared analysis and is consistent with the ordered conformations expected by the extraction treatment we used.^47^ Sample GG100 showed bands related to this polysaccharide matrix, with main bands with maxima at 1410 cm^–1^ related to the symmetrical carboxyl group stretching, and at 1087 cm^–1^ related to symmetrical stretching of the C–O–C bonds.^48^ As the composition of the samples is changed, the spectral profile changes, with a gradual mixing of the signals related to each matrix according to the proportion of each one, which is more evident in the intermediate sample, GGSS_5050. This confirms the presence and mixing of GG and SS in the composite films.

To evaluate the thermal stability of the composite materials, the TGA curves are shown, with the thermal degradation of the films characterized by evident rates of mass loss (Figure 2a). In the first stage, a gradual loss of an average of 15% in mass was detected up to around 200 °C, attributed to water evaporation and loss of low-temperature volatile compounds.^38^ The lower the loss, the higher the proportion of SS (12% for GGSS_1090); SS100 had a different behavior, with a more linear evolution of the decrease.

**Figure 2:**
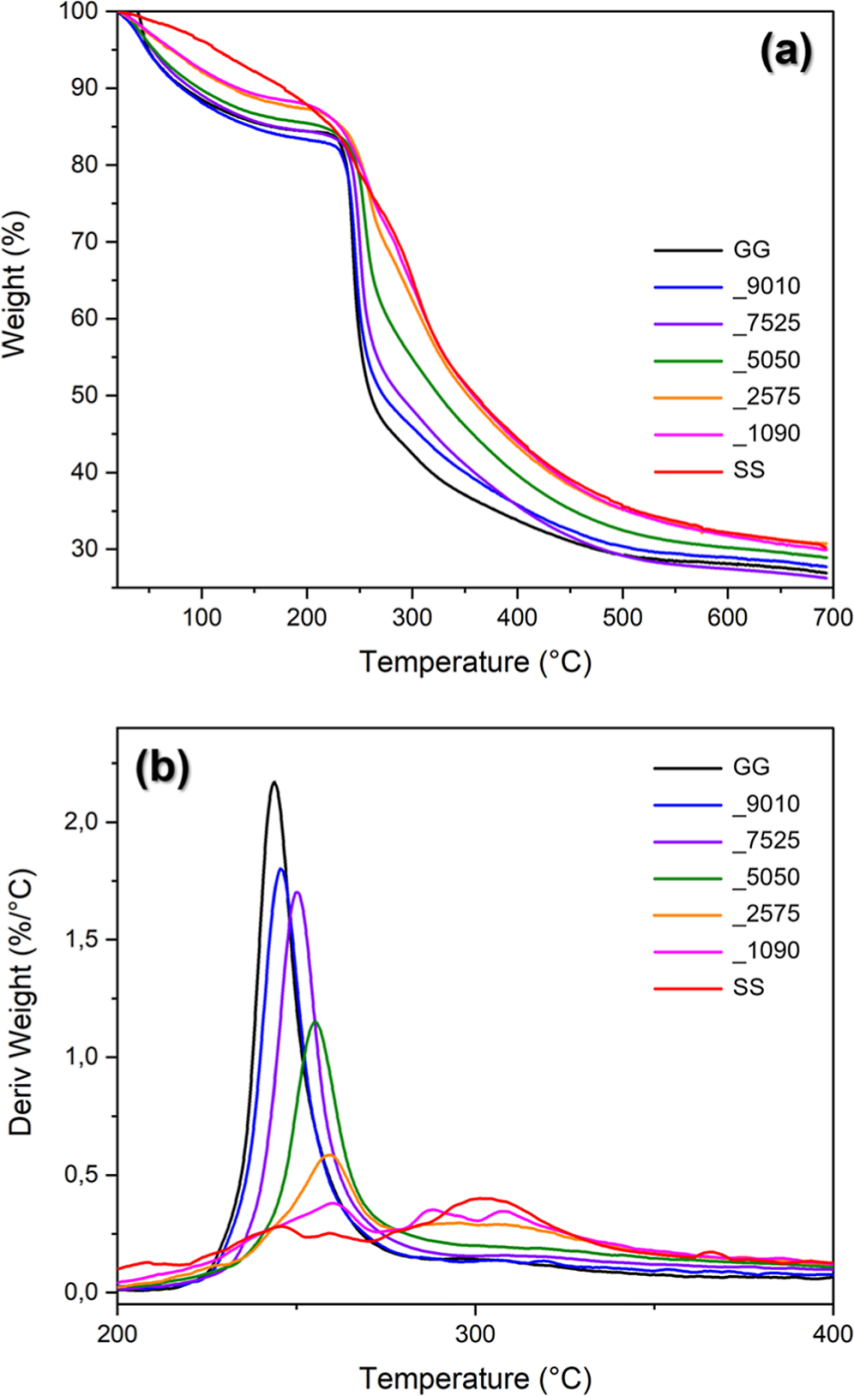
(a) TGA curve and (b) TGA curve derivative in thermogravimetric analysis of GG and SS composite and pristine films.

From 200 °C onward, the different behaviors become more evident, with samples with a greater part of GG losing mass abruptly, as shown in the weight derivative curves (Figure 2b), with a maximum of 2.2%/°C at 243 °C for the GG100. As the proportion of SS increases, this value decreases, with the maximum occurring at a higher temperature, for example, 1.1%/°C at 255 °C for GGSS_5050. In addition to this phenomenon, from GGSS_2575, it is also possible to observe a broad band in the region of 300 °C, which intensifies in SS100, characteristic of this matrix.

Subsequently, the loss of mass becomes continuous, occurring at lower temperatures with GG and at higher temperatures with SS. Concerning SS, from 200 °C onward, losses are attributed to the breaking of side chain groups of amino acid residues and the cleavage of peptide bonds.^49^ For comparison, the residual mass at 300 °C was between 42.5% (GG100), 54.9% (GGSS_5050), and 65.3% (SS100), demonstrating the better thermal stability caused by SS.

Tensile analyses indicated a tendency for Young’s modulus (Table 1) to increase with a higher proportion of GG in the composite matrix, from 1990 MPa (GGSS_5050) to 2426 MPa (GGSS_9010), which indicates an improvement in the property mechanics in films containing SS, becoming more resistant to elastic deformation when stresses are applied.

**Table 1:** Young’s Modulus of Tensile Tests of GG and SS Composite Films

sample	Young’s modulus (MPa)	standard deviation
GGSS_5050	1644	285
GGSS_7030	2100	164
GGSS_9010	2426	235

The composite material presented several physicochemical properties particular to the two matrices, being proportionally changed according to the quantity of each of them. This makes it possible to obtain materials with different transparencies, thermal and mechanical resistances, for example, adjustable according to the intended application.

### 3.2 Soft Lithography Diffraction Patterns

SS films were obtained by casting on the template with submicron-structured patterns, and it was able to reproduce the relief on its surface, which caused the iridescence effect, as shown in Figure 3 (above). The same effect was already observed by Perry^50^ for fibroin films, a fibrous protein with a structural role in silk filaments, which produced films with nano or micropatterns. However, herein, the films produced only based on SS were fragile, brittle, and difficult to handle.

**Figure 3:**
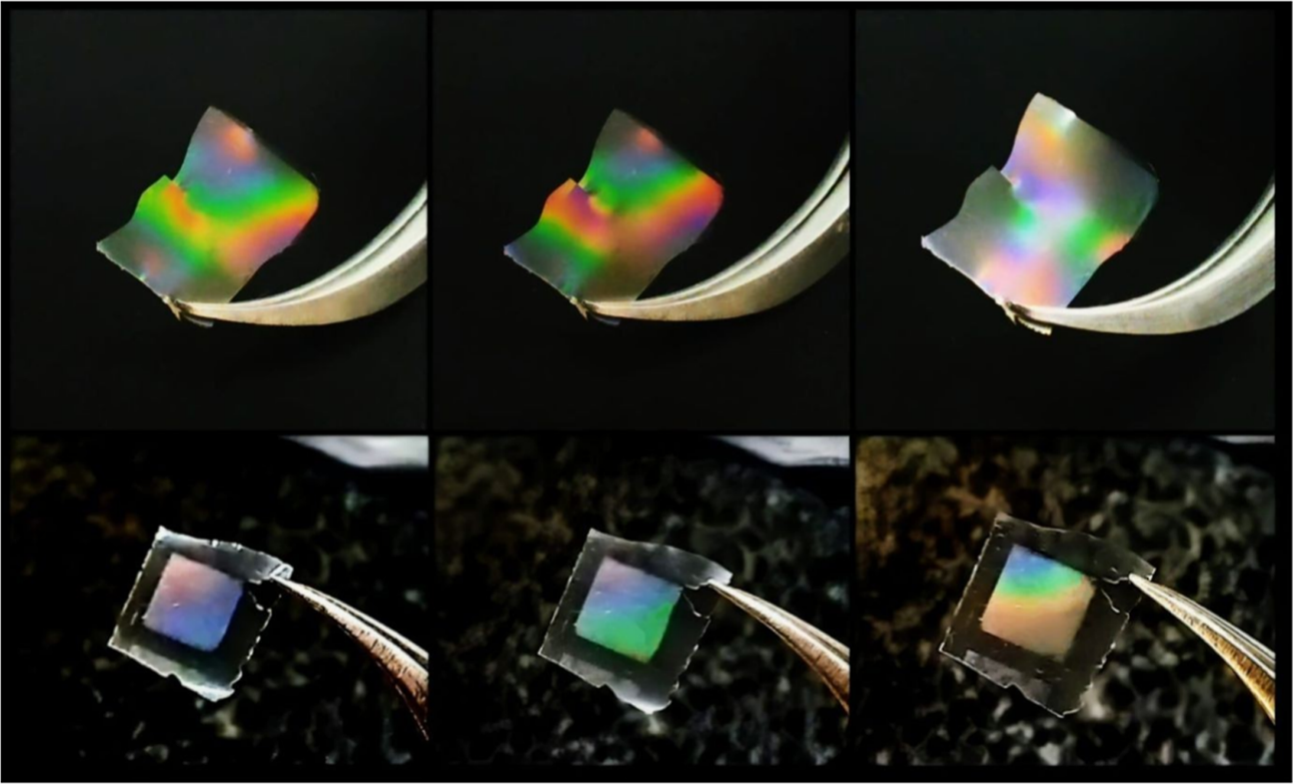
Photograph of (above) SS100_G and (below) GGSS_5050_C films shows the iridescence effect depending on the angle of incidence of light.

As discussed in tensile analysis, composites SS–GG proved to be an efficient way to improve the mechanical properties of the SS films, leading to more elastic final composites. So, the several GGSS ratios were also evaluated on film production by soft lithography (Figures 3 (below) and 4(c–f)). Well-structured films were carried out independent of the GGSS ratio, and the characteristic iridescence resulting from the surface structure was noted for all films. In fact, the mechanical properties from composite films resulted from the synergism of both biopolymers, whereas only the GG solution did not produce structured films, and none reproduced the iridescent effect, so the final structured surface effect on films was conditional to the SS structure.

**Figure 4:**
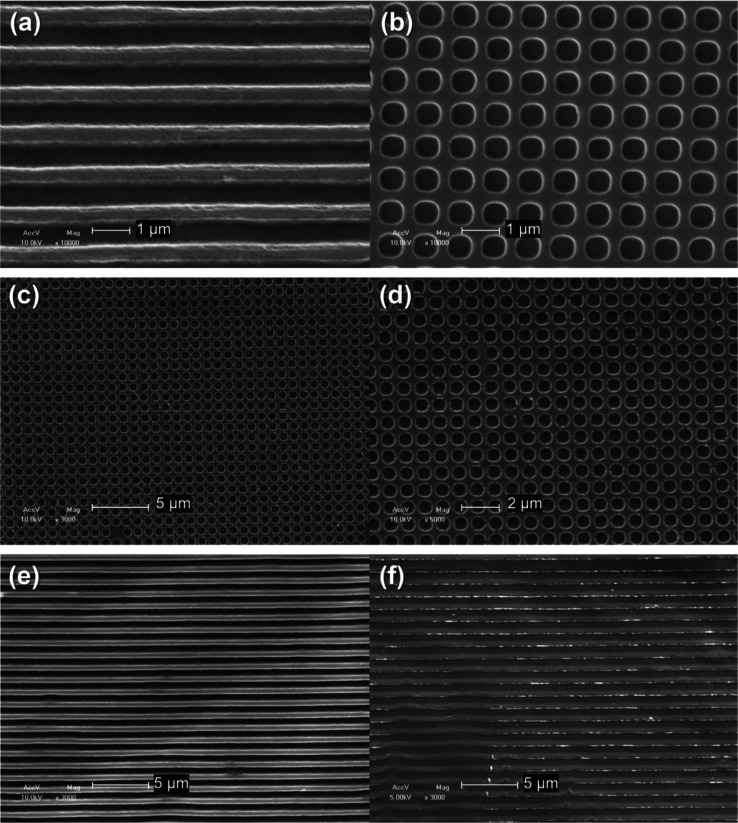
SEM microscopies of the SS100_G (a) and SS100_C (b) films; of the film GGSS_5050_C at different magnitudes (c,d); and films GGSS_1090_G (e) and GGSS_9010_G (f).

The SEM images (Figure 4) confirm that the film kept the structured patterns from the template. The obtained films on mold C presented a surface with periodically ordered circular holes throughout the film, with diameters of approximately 700 nm. Those films carried out on mold G showed ordered channels, 700 nm apart. These periodic patterns at the nanometer scale were responsible for the phenomenon of diffraction of the incident light and resulted in the iridescence effect. To evaluate the regularity of the lithography throughout the film compositions, we measured from these microscopies the diameters of the circular cavities formed in the films with a _C pattern, as well as the lengths of the regions between the circles (edge). Considering all the samples, the diameters varied from 653 to 702 nm and the edges from 268 to 288 nm, with the measurements presenting only a deviation of 23 nm for the circle and 10 nm for the edge, showing that the variation in the size of the micropatterns was minimal considering the scale we are observing, making the effects of lithography independent of the material composition, as long as SS is present.

The shape and depth of these cavities were determined by AFM microscopy (Figure 5). The data from GGSS_5050_C revealed the film surface with circular periodic cavities. Surface roughness values were obtained from the analyzed areas, being amplitude parameters that quantitatively characterize the film topography, which presented “*R*_a_” (an average of the height variations in relation to a mean line) of 31.4 ± 3.8 nm, indicating a nonsmooth surface, and “*R*_z_” (an average of the difference between the highest peak and the deepest valley) of 267.5 ± 19.4 nm, which provides an approximation of the depth of the circular cavities formed by lithography.^51^

**Figure 5:**
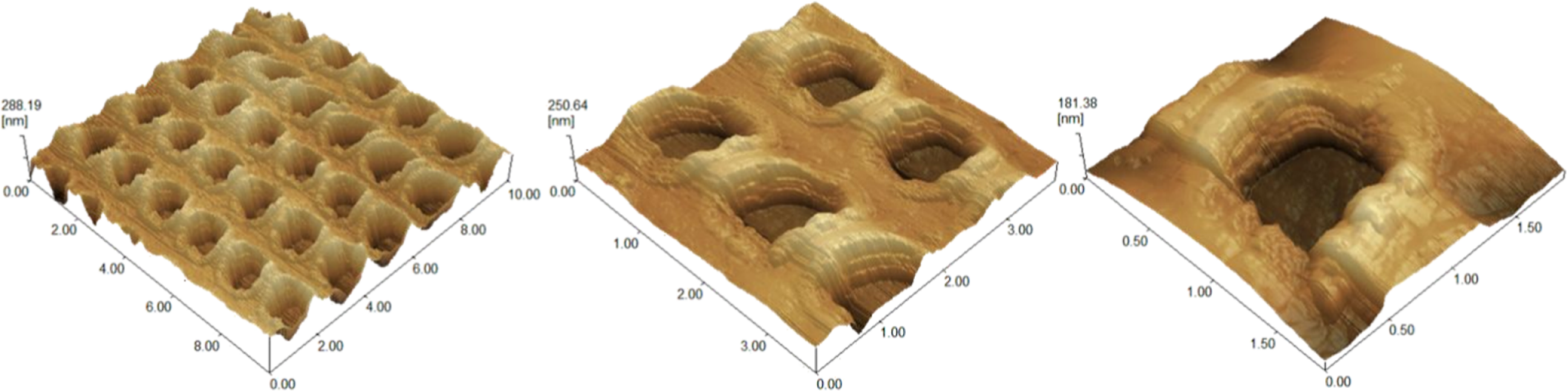
AFM microscopies of sample GGSS_5050_C showing the film surface at various magnifications (10.0 × 10.0 μm, 4.0 × 4.0 μm, and 2.0 × 2.0 μm).

In fact, GG contributed to improve the mechanical properties of the films, which at the same time maintained the inherent property of SS to reproduce patterned surfaces. Only the combination of these two biopolymers collaborates for the formation of a resistant patterned film.

### 3.3 Photoluminescence Properties

Pristine films and composites doped with lanthanide ions were characterized by photoluminescence spectroscopy. The Eu^3+^ ion was used as a spectroscopic structural probe to study the interaction of matrices, and to characterize the interesting emission of the materials. The excitation spectrum for the Eu-doped SS films showed a broad and intense band in the ultraviolet region, with the maximum band at 305 nm (Figure 6a), attributed at the energy transfer from the matrix to Eu^3+^ ion, based on previous work on fibroin, that band could be related to the absorption of amino acids from the SS protein, as the aromatic amino acids Trp and Tyr.^52^

**Figure 6:**
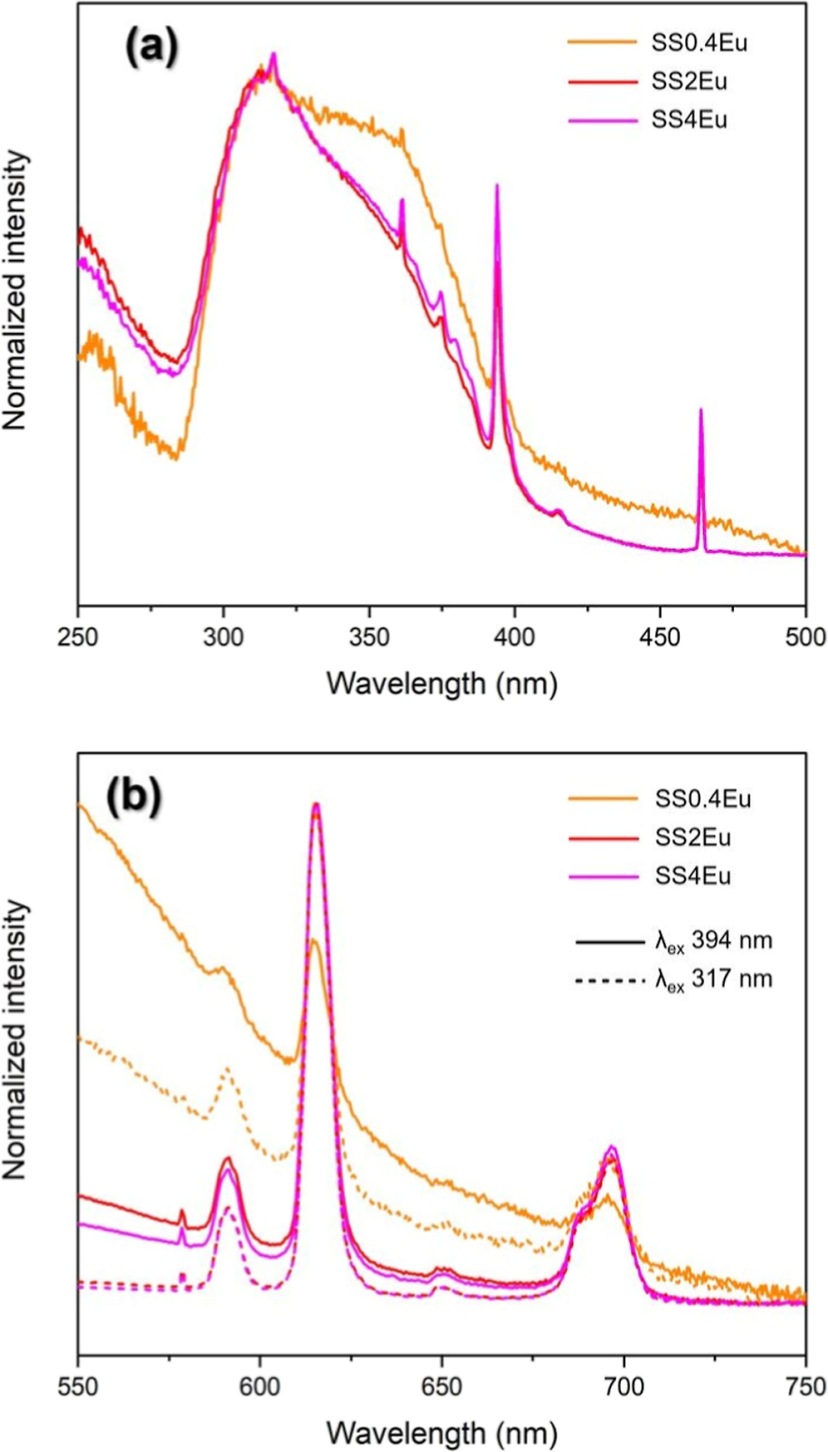
Excitation (λ_em_ = 614 nm) (a) and emission (b) spectra at two wavelengths of the samples SS0.4Eu, SS2Eu, and SS4Eu at room temperature.

The emission spectra showed profiles corresponding to the ion in a low-symmetry environment, with the band at 614 nm referring to the ^5^ *D*_0_ → ^7^ *F*_2_ transition broadened in an inhomogeneous way^32^ and associated with the presence of the transition ^5^ *D*_0_ → ^7^ *F*_0_. The lifetimes for the ^5^ *D*_0_ excited state obtained (monitoring the ^5^ *D*_0_ → ^7^ *F*_2_ transition) were approximately twice those observed for the previously studied GG matrix (τ = 0,34 ms, λ_ex_ 394 nm) doped with the same proportion of Eu^3+^. The values were 0.510 ms (λ_ex_ = 394 nm), 0.660 ms (λ_ex_ = 303 nm), and 0.692 ms (λ_ex_ = 256 nm). These values were coherent with the Eu^3+^ ion coordinated within a hydrophobic portion in the protein structure; similar results were also observed for the fibroin-doped Eu^3+^ ions.

Since the Gd^3+^ ion does not exhibit emissions in the visible region, it was used in the preparation of a film (SS2Gd) to mimic the lanthanide coordination interaction into the SS structure without interference from the ions in the emission spectra. So, it was possible to study emissions originating only from the SS matrix while accounting the disturbance of Ln^3+^ ions on its structure. According to the literature, the singlet and triplet states for the aromatic amino acids constituting the proteins SF and SS are, respectively, Trp 304 and 416 nm, Tyr 287 and 347 nm, and Phe 269 and 341 nm.^53^ The percentage of these amino acids present in the proteins SF and SS is Trp 0.5% (SF) and 0.5% (SS), Tyr 11.8% (SF) and 4.9% (SS), and Phe 1.2% (SF) and 0.6% (SS).^54^ In general, regarding SF, SS has a lower amount of aromatic amino acids in its composition. SF is composed of 13.5% of these amino acids, while SS contains only 6%. However, based on the calculated lifetimes for the Eu^3+^ ions and the spectral profiles, we can conclude that, despite being present in lower concentrations, the aromatic amino acids act as sensitizers for Ln^3+^ ions in the SS matrix through the antenna effect, like what was observed for fibroin. Additionally, akin to the observations for fibroin, through the spectrum deconvolution (Figure 7), it was possible to conclude that the energy transfer occurs preferentially via Trp.^52^ The broad band between 320 and 575 nm, centered at 450 nm, aligns with the energy of the triplet state of the Trp amino acid, as reported in the literature (24,050 cm^–1^) at 77 K.^53^ In summary, since sensitization occurs preferentially via Trp amino acids, and SF and SS contain the same proportion of this amino acid (0.5%), the sensitization of Ln ions can occur similarly in both matrices.

**Figure 7:**
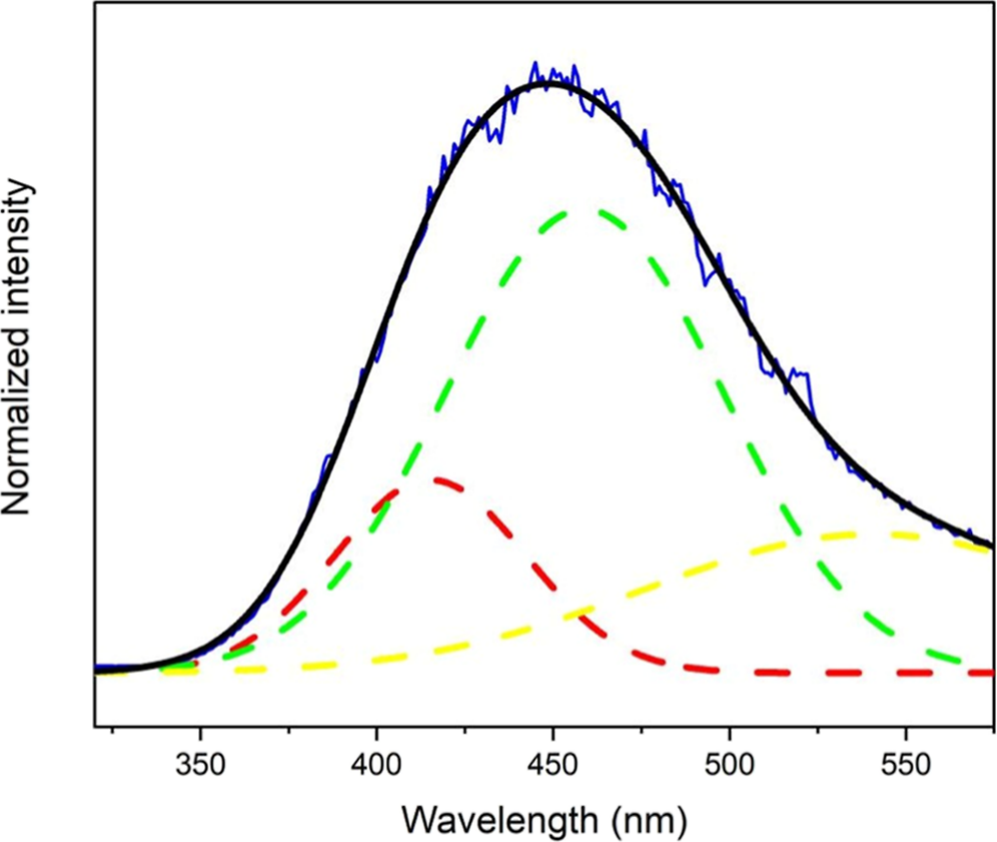
Emission spectra (λ_ex_ = 614 nm) of the sample SS2Gd at 77 K resolved in time (blue line), simulated spectrum (black line), and deconvoluted bands (dashed lines).

For the composite samples of GG and SS doped with Eu^3+^, the addition of Eu^3+^ ion was done by two different ways intended to induce a preferential coordination site, either on GG or on SS. Emission spectra showed similar profiles independent of the preparation method (Figure 8b). However, excitation spectra indicated different behaviors. The samples where europium was added to the GG solution showed an excitation spectrum with a broad band with a maximum at 318 nm, but the intensity was slightly larger than the characteristic f–f band at 394 nm, indicating reduced energy transfer efficiency (Figure 8a). The samples were prepared with the addition of Eu^3+^ to the SS solution, and the excitation spectrum was comparable to the SS_1Eu sample, with an intense and broad band at 305 nm and the relativity intensity was greater than the intra4f6 excitation at 394 nm, indicating that the preparation route of the materials strongly influenced their spectroscopic properties (Figure 8a). The lifetimes for the excited emitter level ^5^ *D*_0_ are described in Table 2.

**Figure 8:**
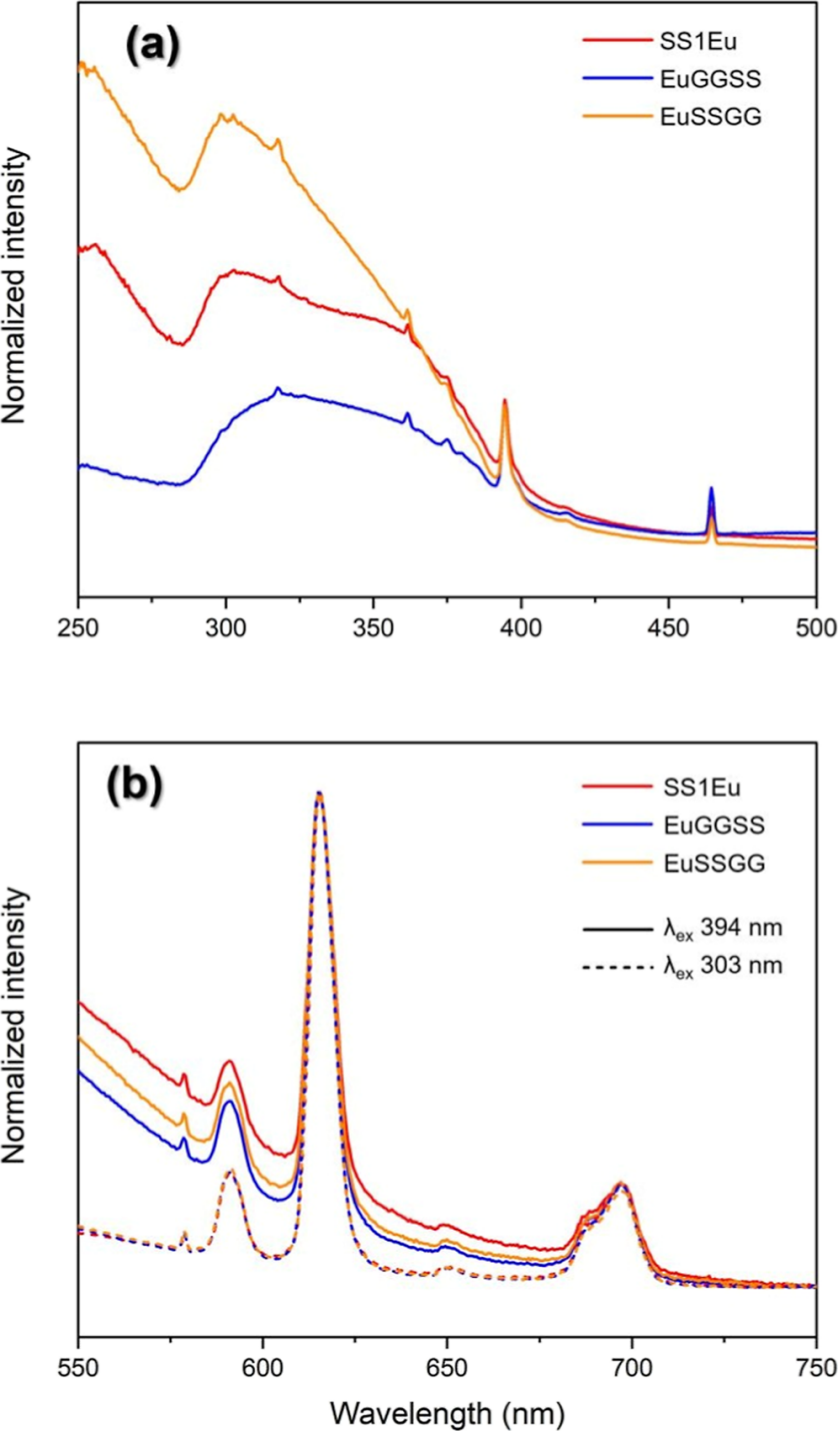
Excitation (λ_em_ = 614 nm) (a) and emission spectra (b) at two wavelengths of the SSEu, EuGGSS, and EuSSGG samples at room temperature.

**Table 2:** Lifetimes (ms) for the Excited Emitter Level ^5^ *D*_0_ (Monitoring the ^5^ *D*_0_ → ^7^ *F*_2_ Transition) in EuSSGG and EuGGSS Samples for the Respective λ_ex_

sample	lifetime (ms)		
EuSSGG	256 nm	303 nm	394 nm
	0.650	0.587	0.482
EuGGSS	280 nm	318 nm	394 nm
	0.713	0.739	0.551

The calculated emission parameters are presented in Table 3. The parameters Ω_2_ and Ω_4_ did not present significant changes between the two kinds of composites (based on the Eu^3+^ addition sequence), as already noted on the spectra, the symmetry characteristics and coordination effects were similar between them. However, the calculated quantum efficiencies were around twice as high for the samples containing SS when compared to the GG_1Eu sample, and the number of water molecules in the first Eu^3+^ coordination sphere decreased from 3 to 1 by the presence of SS in the samples.

**Table 3:** Total Spontaneous Emission Coefficient (*A*_total_), Judd–Ofelt Experimental Intensity Parameters (Ω*n*), Radiative Lifetime (τ_rad_), Experimental Lifetime (τ_exp_), Quantum Efficiency (η), and Number of Molecules of Water in the First Coordination Sphere of the Samples Excited at 394 nm^35,36^

sample	***A***_**total**_ **(ms** ^**‑1**^ **)**	**Ω**_**2**_ **(×10** ^**‑20**^ **cm** ^**2**^ **)**	**Ω**_**4**_ **(×10** ^**‑20**^ **cm** ^**2**^ **)**	**τ**_**rad**_ **(ms)**	**τ**_**exp**_ **(ms)**	η (%)	*q*
GG_1Eu	394.04	6.60	4.59	2.54	0.30	11.8	2.92
SS_1Eu	419.72	7.56	4.23	2.38	0.51	21.4	1.37
EuGGSS	427.64	7.81	4.31	2.34	0.55	23.6	1.20
EuSSGG	414.37	7.50	4.17	2.41	0.48	20.0	1.50

The final coordination environment of the Eu^3+^ ion, as observed from the emission spectra, is notably similar across all composites. However, the composite structure enhances the excitation process through energy transfer from the SS or GG matrices, resulting in an extended lifetime for Eu^3+^ ions within a hydrophilic structure.

## 4 Conclusions

Our results highlight that composites of silk sericin (SS) and gellan gum (GG) offer considerable promise and versatility for photonic applications. The developed self-supporting SS–GG films, prepared through a straightforward high-temperature and high-pressure extraction method, effectively replicate micro- and nanoscale patterns, demonstrating significant potential for advanced biobased photonic devices. The films exhibit favorable mechanical properties due to the inclusion of GG, and their optical properties, such as transparency, can be finely tuned by varying the SS content. Furthermore, doping the composite films with Eu^3+^ ions not only enhanced their optical functionality but also provided insights into the specific interactions between lanthanide ions and the biopolymer matrices. Our findings highlight SS and GG as promising, underexplored materials in photonics, significantly expanding the scope of biobased materials in optical applications, such as exploring these advantages to materials such as OLED devices, films for smart packaging, and random laser systems. This work thus contributes to advancing the field of sustainable photonics by introducing innovative silk-based composites as versatile platforms for sensors, biointegrated devices, and photonic technologies.

## Data Availability

All relevant data is available from the authors.
